# The Association Between Cytomegalovirus Infection and Increased Leukemia Incidence: A Retrospective Matched Cohort

**DOI:** 10.7759/cureus.95614

**Published:** 2025-10-28

**Authors:** Ernesto Joubran, Lily Tehrani, Leah Ilyaev, Michelle Tashjian, Aneil Walizada, Manell Aboutaleb, German Giese

**Affiliations:** 1 Medicine, Nova Southeastern University Dr. Kiran C. Patel College of Osteopathic Medicine, Fort Lauderdale, USA; 2 Pain Management and Rehabilitation, Nassau University Medical Center, East Meadow, USA; 3 Internal Medicine, HCA Florida Westside, Plantation, USA; 4 Family Medicine, Henry Ford Health System, Detroit, USA; 5 Internal Medicine, University of Miami Miller School of Medicine at Holy Cross Hospital, Miami, USA

**Keywords:** acute myeloid leukemia, cytomegalovirus infection, epidemiology, hematologic malignancy, leukemogenesis, natural killer cells, oncogenic viruses, viral immunotherapy

## Abstract

Introduction

Cytomegalovirus (CMV) is a common viral infection with high global seroprevalence that often remains dormant throughout life. While typically asymptomatic, early exposure has been linked in some studies to hematologic malignancies, including acute myelogenous leukemia (AML). Evidence suggests CMV may play both protective and contributory roles in leukemogenesis. This retrospective study evaluates the correlation between CMV infection and the incidence of leukemia in a national cohort.

Methods

This retrospective matched cohort study used data from a Health Insurance Portability and Accountability Act (HIPAA) compliant national database (PearlDiver Marina, PearlDiver Technologies Inc., Colorado Springs, CO) using International Classification of Diseases (ICD)-9 and ICD-10 codes. Inclusion required at least eight years of continuous active status. Controls were matched 1:1 to CMV patients by age, sex, and Charlson Comorbidity Index (CCI). Statistical analyses were conducted using Pearson’s chi-square tests, with odds ratios (ORs), relative risk (RR), and 95% confidence intervals (CIs) reported. Two-tailed p-values < 0.05 were considered significant.

Results

Between January 2010 and December 2019, 14,268 patients with CMV infection were matched 1:1 to 14,268 controls. The incidence of leukemia was 2.61% (373/14,268) in the CMV group and 1.56% (224/14,268) in controls (RR 1.66, 95% CI 1.41-1.94; OR 1.69, 95% CI 1.44-1.97; p < 0.001). A temporally restricted analysis isolating incident leukemia cases following CMV exposure demonstrated a statistically significant correlation between CMV infection and leukemia.

Conclusion

Prior CMV infection was associated with an increased risk of developing leukemia in subsequent years, supporting the need for additional studies to validate these findings and identify the underlying mechanisms of CMV-related hematologic malignancy.

## Introduction

Leukemia

Leukemia encompasses a broad group of aggressive blood and bone marrow malignancies, classified by tempo (acute vs. chronic) and lineage (myeloid vs. lymphoid). The American Cancer Society and Surveillance, Epidemiology, and End Results (SEER) program estimate that there were approximately 60,650 new leukemia cases and 24,000 deaths in the U.S. during 2022, with about 20,050 of these new cases categorized as acute myeloid leukemia (AML) [1,2].

Protective effects of CMV

Given its immunomodulatory properties, cytomegalovirus (CMV) has been studied in this context due to its ability to activate natural killer (NK) cell subsets and augment anti-leukemic activity [3]. NK cells are key mediators of graft-versus-leukemia after hematopoietic cell transplantation (HCT), and adoptive/allogeneic NK strategies have shown potential to induce remission and reduce relapse across hematologic malignancies [4-6]. The effectiveness of NK-based responses is reduced when tumor cells express HLA-E, which engages the inhibitory NK receptor NKG2A [7]. By contrast, NK cells expressing the activating receptor NKG2C can lyse HLA-E-positive tumors, though NKG2C⁺ NK cells are relatively infrequent in healthy adults [8,9]. CMV reactivation after allo-HCT has been associated with a reduced relapse risk in AML [10,11], and this protection has been linked to expansions of adaptive “memory-like” NK phenotypes, including NKG2C⁺ subsets [12,13]. Even outside the transplant setting, CMV seropositivity has been associated with enhanced NK cytotoxicity against hematologic targets, with activity modulated by tumor HLA-E and donor NK phenotype [14,15].

Epidemiologic signals linking CMV and leukemia

While some data suggest that CMV may confer context-dependent protection, other evidence raises concerns about leukemogenic associations. Despite these potential anti-leukemic effects, emerging data indicate that CMV may also play a pathogenic or oncogenic role under certain conditions. In a large population-based analysis, early CMV infection, particularly maternal CMV during pregnancy, was associated with increased risk of childhood hematologic malignancies [16]. Similarly, in utero CMV infection has been associated with an increased risk of childhood acute lymphoblastic leukemia (ALL) [17].

CMV, oncomodulation, and oncogenesis

Beyond epidemiology, mechanistic and translational work suggests CMV may influence tumor biology. Michaelis, Doerr, and Cinatl advanced the concept of “oncomodulation,” in which CMV infection of tumor or stromal cells enhances malignant traits (e.g., invasiveness, dysregulated signaling) without de novo transformation [18]. Soroceanu and Cobbs reviewed evidence that CMV perturbs tumor-suppressor pathways, mitogenic signaling, angiogenesis, and immune evasion, potentially promoting tumor progression [19]. Herbein further synthesized data indicating that specific CMV-encoded proteins may possess intrinsic oncogenic potential and, in experimental systems, can drive tumorigenic programs in vivo [20].

Study objective

This retrospective cohort study evaluates the correlation between prior CMV infection and the incidence of leukemia in a large, nationally representative population, testing the hypothesis that documented CMV exposure is associated with a higher likelihood of subsequent leukemia diagnosis compared with no prior CMV exposure.

## Materials and methods

Study design and data source

This retrospective cohort study utilized a Health Insurance Portability and Accountability Act (HIPAA)-compliant national database (PearlDiver Marina, PearlDiver Technologies Inc., Colorado Springs, CO), accessed through the Bellwether interface. The database contains over 41 billion de-identified patient records derived from private insurance claims, including Humana, UnitedHealthcare, and Medicare, as well as commercial plans, Medicaid, and self-pay populations. Because all data were de-identified and publicly available, Institutional Review Board (IRB) approval was not required.

The study population comprised patients with active database status between January 2010 and December 2019, and data were queried in May 2022. International Classification of Diseases, Ninth and Tenth Revisions (ICD-9 and ICD-10) and Current Procedural Terminology (CPT) codes were used to identify patients diagnosed with CMV infection and leukemia within the PearlDiver database via the Bellwether statistical interface, which automatically applies de-identified patient identifiers to prevent duplication across payers.

Inclusion and exclusion criteria

Inclusion criteria required at least eight years of continuous “active” status within the database to ensure sufficient longitudinal follow-up. Patients lacking a suitable match were excluded to maintain balanced cohorts. In the secondary (temporally restricted) analysis, patients diagnosed with leukemia before CMV infection were excluded to preserve directionality. For control patients, the index date was assigned as the matched CMV patient’s diagnosis date to maintain temporal alignment.

Cohort matching

Two cohorts were established: a CMV-positive group (patients with a history of CMV infection) and a control group (patients without such a history). To minimize confounding bias, cohorts were propensity-matched 1:1 by age range, sex, and Charlson Comorbidity Index (CCI) using the PearlDiver matching algorithm. The CCI is a validated measure estimating one-year mortality risk based on comorbidities and provides a standardized adjustment for disease burden [21]. Patients without an appropriate match were excluded. All analyses were performed on the same 1:1 propensity-matched cohort of 14,268 CMV-positive and 14,268 CMV-negative individuals.

The primary (unrestricted) analysis included any leukemia diagnosis, regardless of timing relative to CMV infection. To evaluate the potential confounding effect of antiviral therapy, matched cohorts were stratified, rather than re-matched, by antiviral exposure, as few control patients received such therapy. The secondary (temporally restricted) analysis included only leukemia cases occurring after the CMV index diagnosis, isolating incident cases and allowing for assessment of temporal association.

Outcome measures 

**Figure 1:**
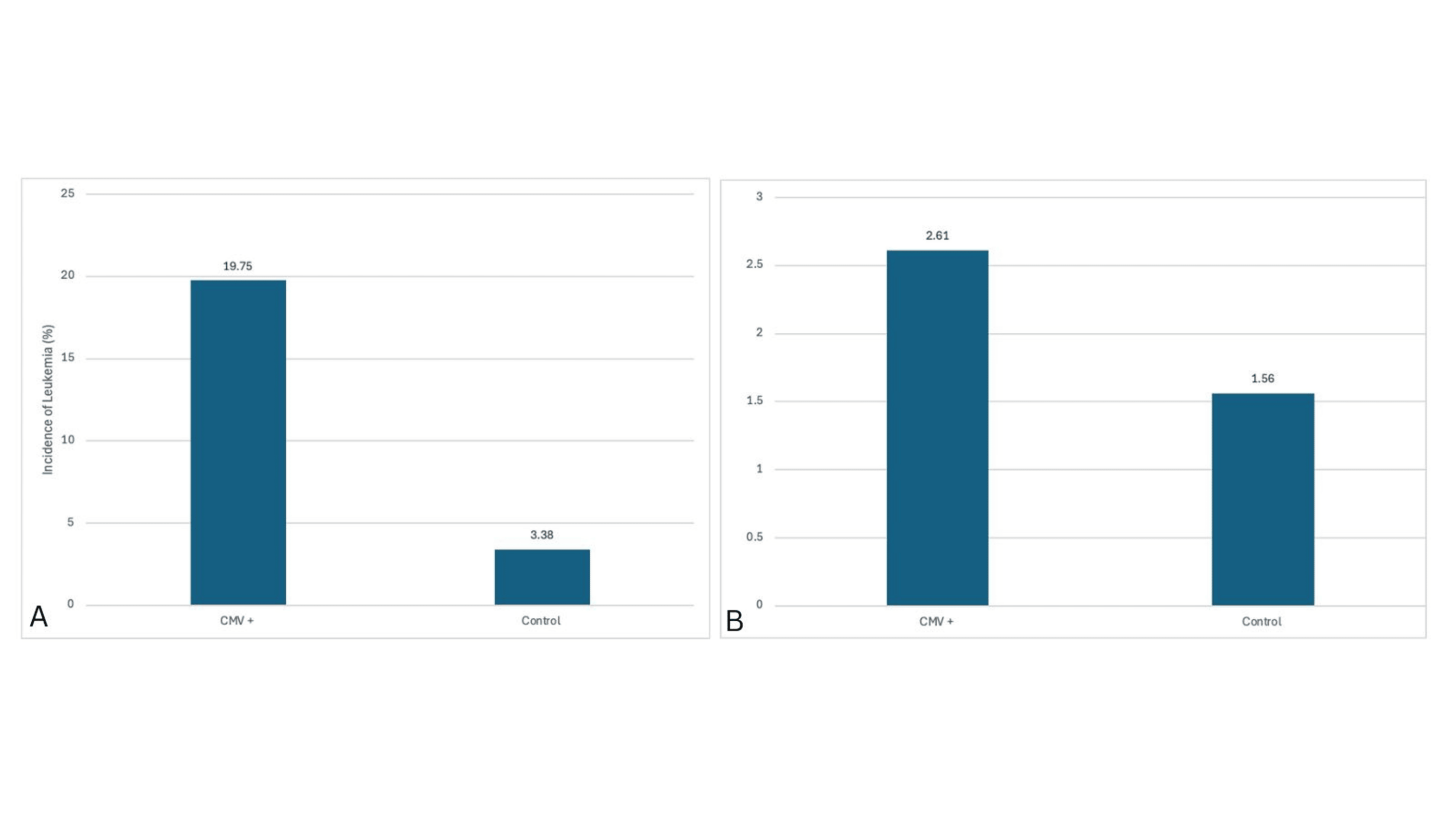
Incidence of leukemia among patients with and without prior CMV infection across unrestricted and temporally restricted analyses. (A) Unrestricted analysis includes all leukemia diagnoses, regardless of timing relative to CMV infection. Among 14,268 CMV-positive and 14,268 matched controls, 2,818 (19.75%) and 482 (3.38%) patients had leukemia, respectively (relative risk (RR) = 5.86, 95% confidence interval (CI) 5.32-6.42; odds ratio (OR) = 7.04, 95% CI 6.37-7.77; p < 2.2 × 10⁻¹⁶). (B) Temporally restricted analysis includes only leukemia diagnoses occurring after CMV infection. In the same matched cohort, 373 (2.61%) CMV-positive and 224 (1.56%) control patients developed incident leukemia (RR = 1.66, 95% CI 1.41-1.94; OR = 1.69, 95% CI 1.44-1.97; p < 0.001). CMV = cytomegalovirus.

The primary outcome was the association between CMV infection and leukemia compared with controls (Figure 1A). This “unrestricted” analysis included any leukemia diagnosis, regardless of whether it occurred before or after CMV infection.

A secondary, temporally restricted analysis (Figure 1B) included only leukemia cases diagnosed after the index CMV diagnosis to better approximate incident disease risk following CMV exposure.

Additional analyses stratified results by age, sex, geographic region, and diagnosis year to assess consistency across subgroups. Following demographic analysis, potential treatment bias was assessed by stratifying patients according to antiviral exposure. Although the control group contained few patients receiving antivirals, this step evaluated whether antiviral use materially influenced the observed association.

Statistical analysis 

All analyses were conducted using PearlDiver Marina statistical software. Descriptive statistics summarized baseline cohort characteristics. Pearson’s chi-square (χ²) tests evaluated post-match balance and compared leukemia incidence between CMV-positive and CMV-negative patients.

From 2 × 2 contingency tables representing leukemia presence or absence in CMV-exposed versus unexposed individuals, the software automatically calculated χ² statistics, odds ratios (ORs), relative risks (RRs), and corresponding 95% confidence intervals (CIs). All hypothesis tests were two-tailed, with statistical significance defined as p < 0.05.

## Results

**Figure 2:**
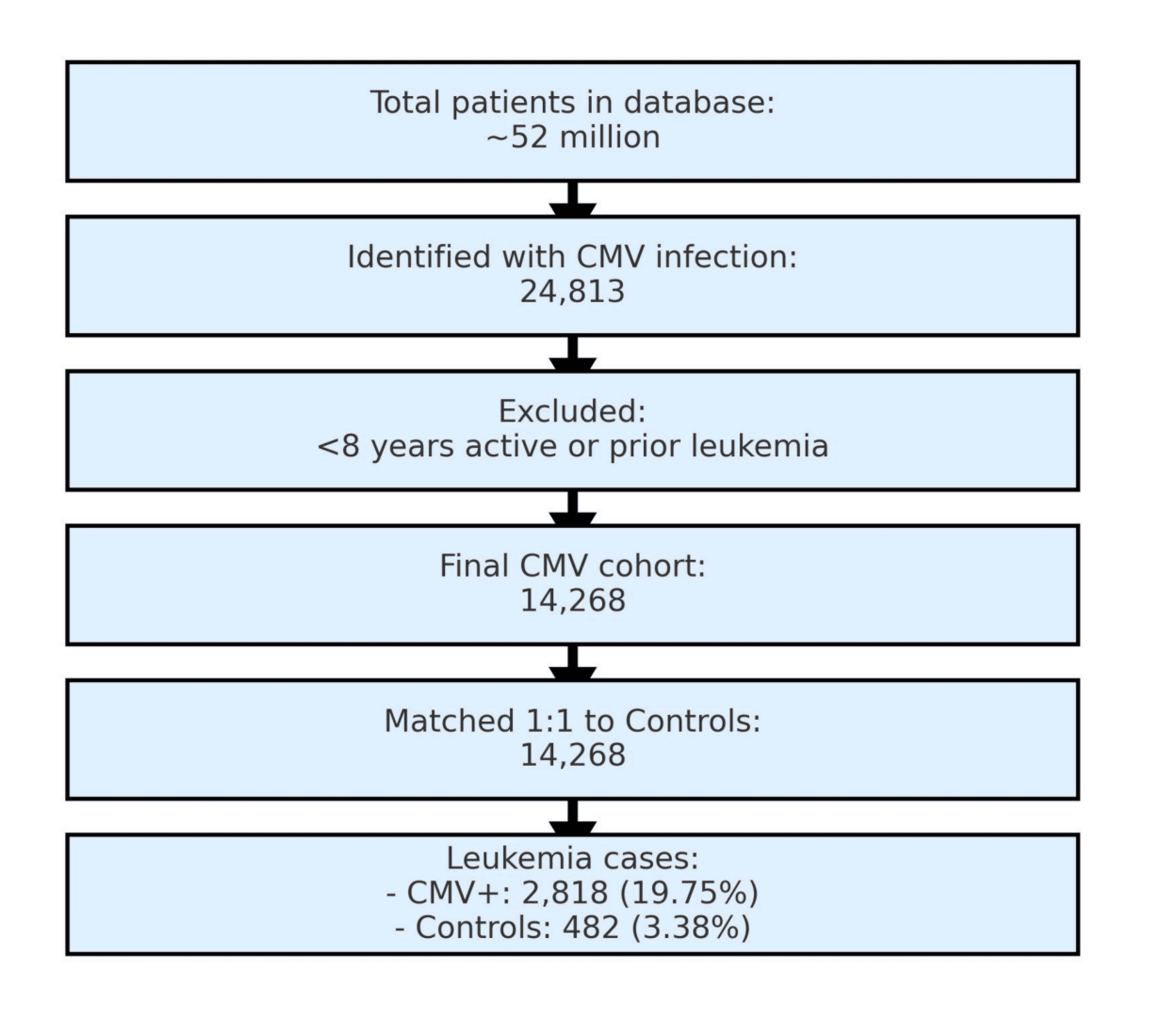
Stepwise progression of patient group selection from the database. From a total of approximately 52 million patients, 24,813 with a history of cytomegalovirus (CMV) infection were identified. After applying eligibility criteria (≥ 8 years active status; exclusion of prior leukemia), 14,268 patients with CMV infection were matched 1:1 with 14,268 controls. Among these matched groups, 2,818 (19.75%) CMV-positive and 482 (3.38%) control patients had leukemia. This diagram illustrates cohort construction for the primary (unrestricted) analysis; the secondary (temporally restricted) analysis (Figure 1B) used the same matched cohort but was limited to leukemia cases diagnosed after CMV infection.

Data analyzed between January 2010 and December 2019 revealed 14,268 patients with CMV infection and an equal number of matched controls. Using a stepwise selection process (Figure 2), we conducted a primary (unrestricted) analysis (Figure 1A) that demonstrated a significant association between CMV infection and leukemia (p < 2.2 × 10⁻¹⁶; OR 7.04, 95% CI 6.37-7.77). This primary analysis included any leukemia diagnosis, regardless of whether it occurred before or after CMV infection.

A total of 2,818 patients with CMV infection were also diagnosed with leukemia at any time point (19.75%), compared with 482 patients without CMV infection (3.37%) (Figure 1A). Individuals with CMV infection had a markedly increased likelihood of a leukemia diagnosis (RR 5.86, 95% CI 5.32-6.42; OR 7.04, 95% CI 6.37-7.77; p < 2.2 × 10⁻¹⁶). This association remained significant after matching for age and CCI. 

**Figure 3:**
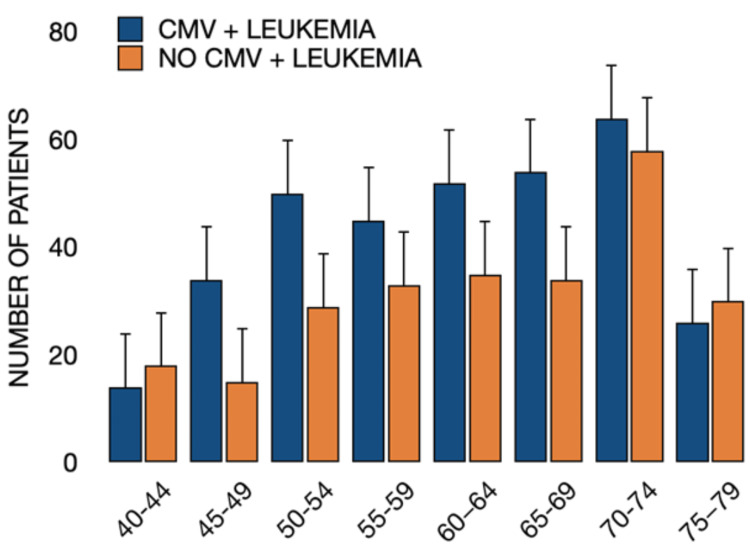
Age distribution of patients diagnosed with leukemia with and without prior CMV infection. Distribution of patients diagnosed with leukemia stratified by age and history of CMV infection. Across most age groups, a higher number of patients with prior CMV infection developed leukemia compared to patients without CMV infection. Comparisons between groups were made using Pearson’s chi-square (χ²) test; p < 0.05 was considered statistically significant. CMV = cytomegalovirus.

**Figure 4:**
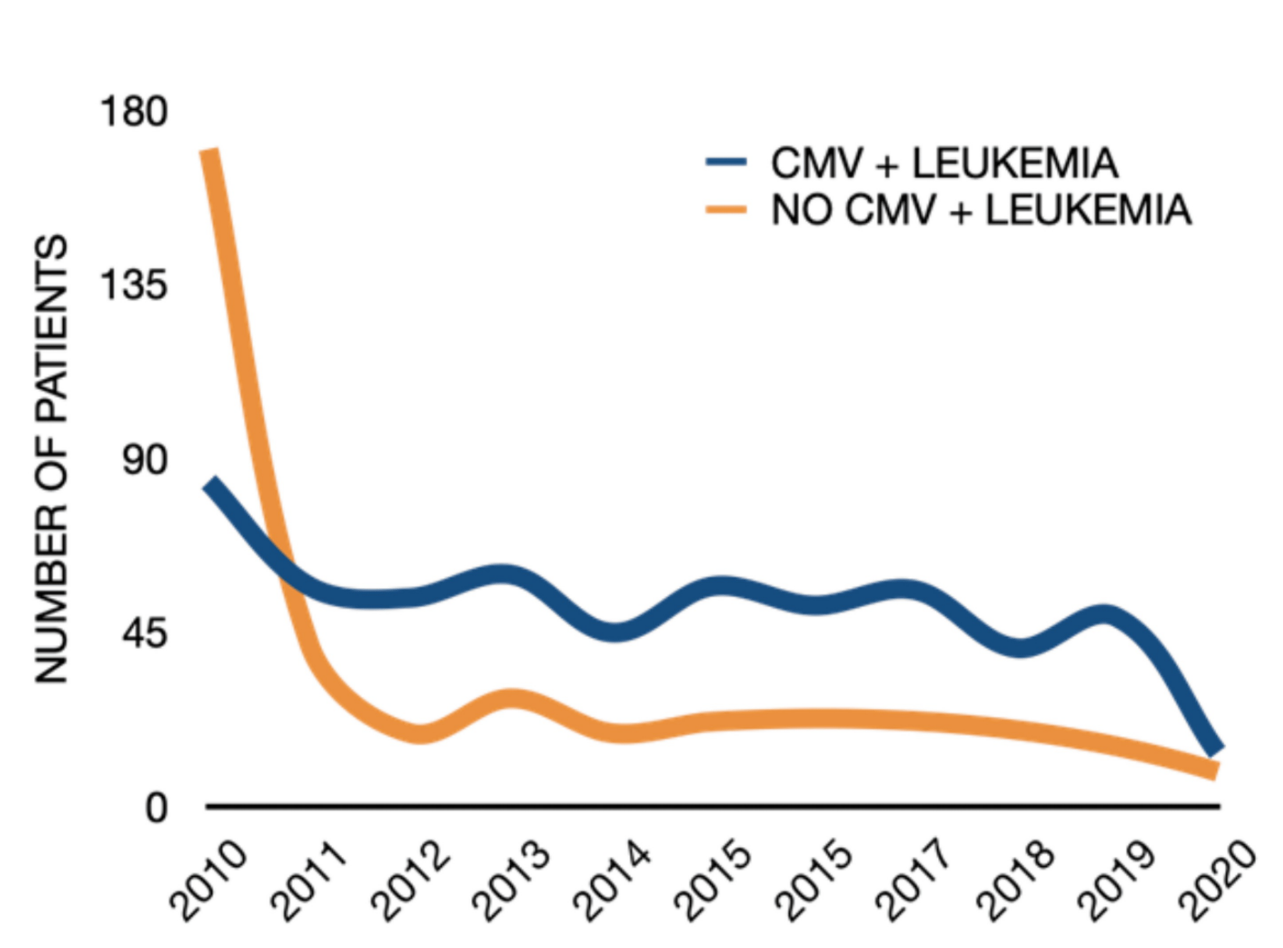
Annual incidence of leukemia in patients with and without prior CMV infection. Trends in the incidence of leukemia from 2010 to 2020 in patients with a history of CMV infection compared to those without prior CMV infection. Across the study period, patients with prior CMV infection consistently demonstrated higher leukemia incidence than those without CMV infection. Statistical comparisons across groups were conducted using Pearson’s chi-square (χ²) test; p < 0.05 (two-tailed) was considered significant. CMV = cytomegalovirus.

As expected, leukemia incidence increased with age, but patients with CMV infection consistently exhibited higher rates of leukemia than CMV-negative controls across all age groups (Figure 3). Age stratification began with the 40-44 group, as younger cohorts contained too few patients for HIPAA-compliant reporting. The highest incidence was observed in the 70-74 age group. Temporal stratification by year of leukemia diagnosis indicated stable rates across the study period, minimizing potential over- or under-diagnosis bias (Figure 4).

**Figure 5:**
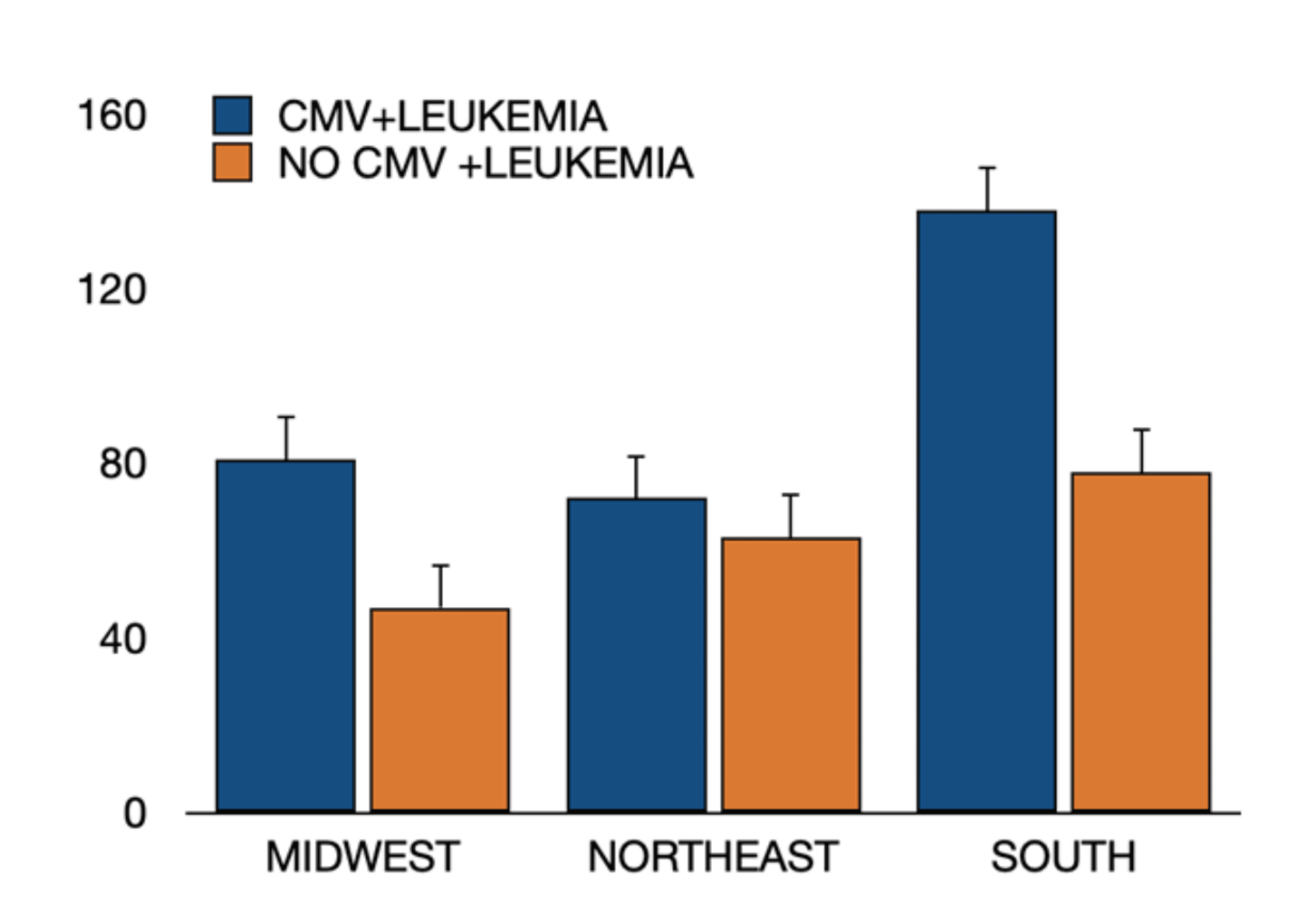
Regional distribution of leukemia cases in patients with and without prior CMV infection. Geographic distribution of leukemia cases across U.S. regions (Midwest, Northeast, and South) among patients with a history of CMV infection compared to those without prior CMV infection. In all regions, patients with prior CMV infection demonstrated a higher incidence of leukemia than non-CMV patients. Differences between groups were evaluated using Pearson’s chi-square (χ²) test; significance was defined as p < 0.05 (two-tailed). CMV = cytomegalovirus.

Geographic stratification revealed higher overall leukemia incidence in the South compared with the Midwest and Northeast; however, the increased risk associated with CMV infection was consistent across all regions (Figure 5).

**Figure 6:**
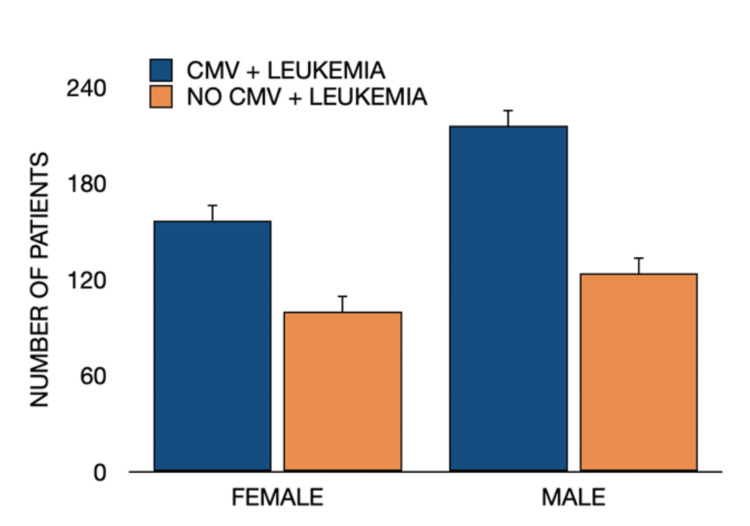
Gender distribution of leukemia cases in patients with and without prior CMV infection. Distribution of leukemia cases stratified by gender among patients with and without a history of CMV infection. Both male and female patients with prior CMV infection demonstrated higher leukemia incidence compared to those without CMV infection. Group comparisons were analyzed using Pearson’s chi-square (χ²) test; two-tailed significance set at p < 0.05. CMV = cytomegalovirus.

Gender analysis showed leukemia was more common in males, yet both males and females with prior CMV infection demonstrated higher incidence rates compared to their CMV-negative counterparts (Figure 6).

To account for potential treatment bias, patients were further stratified based on antiviral medication exposure. Although limited by the small number of controls who received antivirals, this subgroup analysis helped assess whether antiviral therapy influenced the magnitude or direction of the CMV-leukemia association.

In the secondary (temporally restricted) analysis (Figure 1B), which included only patients who developed leukemia after their CMV index diagnosis, the incidence of leukemia was 2.61% (373/14,268) in the CMV group and 1.56% (224/14,268) in controls (RR 1.66, 95% CI 1.41-1.94; OR 1.69, 95% CI 1.44-1.97; p < 0.001). By isolating incident leukemia cases following CMV exposure, this analysis produced a smaller but still statistically significant correlation, reinforcing that CMV infection remains an independent predictor of subsequent leukemia diagnosis.

## Discussion

This retrospective analysis of a large national database identified a statistically significant association between CMV infection and leukemia. Using both unrestricted and temporally restricted analytic frameworks (Figures 1A-1B), we found that CMV infection was strongly associated with leukemia overall, and this relationship persisted even when the analysis was limited to cases in which leukemia developed after CMV infection. Patients with a documented history of CMV had a higher likelihood of being diagnosed with leukemia compared to matched controls, and this association persisted after adjusting for age, gender, geography, and comorbidity burden. The temporally restricted analysis yielded a smaller but still significant effect size, reinforcing that CMV exposure preceded and may contribute to subsequent leukemia development. These results align with emerging data on virus-associated hematologic malignancies and underscore the need to explore CMV as a potential cofactor in leukemogenesis.

Our findings are consistent with prior research noting that CMV reactivation correlates with differences in leukemia outcomes. In post-transplant settings, CMV positivity has been linked to lower relapse rates in acute myeloid leukemia, possibly reflecting immune activation and expansion of adaptive NKG2C⁺ NK-cell subsets with enhanced cytotoxicity [10-13]. Conversely, large epidemiologic studies have associated early CMV infection, particularly maternal or in utero exposure, with increased risk of hematologic malignancy [16,17]. Together, these observations suggest that CMV’s relationship with leukemia may be bidirectional and context-dependent, potentially protective when reactivation triggers immune surveillance in immunocompromised hosts but associated with heightened baseline susceptibility in immunocompetent populations.

Biological mechanisms 

Several studies have demonstrated biologically plausible mechanisms that may explain this association. CMV infection of CD34⁺ hematopoietic progenitor cells has been shown to trigger the ERK/MAPK signaling cascade and up-regulate the anti-apoptotic protein MCL-1, enabling survival of latently infected progenitors [22]. Other investigations report that CMV sustains basal epidermal growth factor receptor (EGFR) signaling in these progenitors, maintaining stem-cell-like characteristics and promoting accumulation of genomic lesions [23]. Additional work indicates that the CMV-encoded chemokine receptor US28 constitutively activates the HIF-1α/PKM2 axis, driving proliferative and metabolic reprogramming in infected cells [24]. Collectively, these findings support a model in which CMV latency alters survival and signaling pathways within hematopoietic progenitors, creating a microenvironment permissive to leukemic transformation.

CMV infection also induces durable changes in host immunity, particularly in NK-cell differentiation and adaptive immune remodeling [3-9,14,15]. While these effects may enhance anti-tumor cytotoxicity in some contexts, chronic immune stimulation and dysregulation can also favor malignant evolution within hematopoietic compartments. The consistent elevation in leukemia incidence across age strata observed in both analyses underscores the importance of age-related immune senescence as a potential amplifier of CMV-associated risk (Figure 3). Future research should examine how CMV-driven immune remodeling interacts with genetic or environmental cofactors to influence leukemogenic potential. Although matching was performed for age, sex, and overall comorbidity burden, potential confounding from unmeasured factors such as immunosuppressive conditions, prior chemotherapy, or other viral infections cannot be excluded.

Clinical and research implications 

From a clinical perspective, these results justify further exploration of CMV as a potential environmental or immunologic cofactor in leukemia development. Although causality cannot be inferred from retrospective data, the persistence of a significant association under both analytic conditions strengthens the evidence for a temporally linked, biologically meaningful relationship. Prospective studies incorporating CMV viral load, latency markers, and longitudinal immune profiling will be essential to determine whether CMV acts as a direct driver, facilitator, or surrogate marker of leukemogenic processes.

## Conclusions

It has remained unclear whether CMV is truly an oncolytic or oncogenic virus. As such, this study aimed to clarify and identify the correlation between the two in the general population, outside of transplant and pediatric settings. By incorporating both unrestricted and temporally restricted analyses, we found that CMV infection was associated with an increased likelihood of leukemia diagnosis, and this association persisted even when limited to cases occurring after CMV infection. While methodological constraints shape interpretation, these results highlight a potential oncogenic role of CMV that warrants further investigation.

Further research is needed to examine the mechanisms underlying this association before such information can be applied to treatment and prevention strategies. Our findings provide an important foundation for generating hypotheses and emphasize the need for prospective studies to clarify the biological and clinical nature of the potential relationship between CMV and leukemia.

## References

[1] (2025). SEER Cancer Stat Facts: Leukemia. National Cancer Institute. https://seer.cancer.gov/statfacts/html/leuks.html.

[2] (2025). Key statistics for acute myeloid leukemia (AML). American Cancer Society. https://www.cancer.org/cancer/types/acute-myeloid-leukemia/about/key-statistics.html.

[3] Cooley S, Parham P, Miller JS (2018). Strategies to activate NK cells to prevent relapse and induce remission following hematopoietic stem cell transplantation. Blood.

[4] Bigley AB, Baker FL, Simpson RJ (2018). Cytomegalovirus: an unlikely ally in the fight against blood cancers?. Clin Exp Immunol.

[5] Miller JS, Soignier Y, Panoskaltsis-Mortari A (2005). Successful adoptive transfer and in vivo expansion of human haploidentical NK cells in patients with cancer. Blood.

[6] Shi J, Tricot G, Szmania S (2008). Infusion of haplo-identical killer immunoglobulin-like receptor ligand mismatched NK cells for relapsed myeloma in the setting of autologous stem cell transplantation. Br J Haematol.

[7] Palmer JM, Rajasekaran K, Thakar MS, Malarkannan S (2013). Clinical relevance of natural killer cells following hematopoietic stem cell transplantation. J Cancer.

[8] Sarkar S, van Gelder M, Noort W (2015). Optimal selection of natural killer cells to kill myeloma: the role of HLA-E and NKG2A. Cancer Immunol Immunother.

[9] Lopez-Vergès S, Milush JM, Schwartz BS (2011). Expansion of a unique CD57⁺NKG2Chi natural killer cell subset during acute human cytomegalovirus infection. Proc Natl Acad Sci U S A.

[10] Elmaagacli AH, Steckel NK, Koldehoff M (2011). Early human cytomegalovirus replication after transplantation is associated with a decreased relapse risk: evidence for a putative virus-versus-leukemia effect in acute myeloid leukemia patients. Blood.

[11] Green ML, Leisenring WM, Xie H (2013). CMV reactivation after allogeneic HCT and relapse risk: evidence for early protection in acute myeloid leukemia. Blood.

[12] Cichocki F, Cooley S, Davis Z (2016). CD56dimCD57+NKG2C+ NK cell expansion is associated with reduced leukemia relapse after reduced intensity HCT. Leukemia.

[13] Nguyen S, Dhedin N, Vernant JP (2005). NK-cell reconstitution after haploidentical hematopoietic stem-cell transplantations: immaturity of NK cells and inhibitory effect of NKG2A override GvL effect. Blood.

[14] Bigley AB, Rezvani K, Pistillo M (2015). Acute exercise preferentially redeploys NK-cells with a highly-differentiated phenotype and augments cytotoxicity against lymphoma and multiple myeloma target cells. Part II: impact of latent cytomegalovirus infection and catecholamine sensitivity. Brain Behav Immun.

[15] Bigley AB, Rezvani K, Shah N (2016). Latent cytomegalovirus infection enhances anti-tumour cytotoxicity through accumulation of NKG2C+ NK cells in healthy humans. Clin Exp Immunol.

[16] Wiemels JL, Talbäck M, Francis S, Feychting M (2019). Early infection with cytomegalovirus and risk of childhood hematologic malignancies. Cancer Epidemiol Biomarkers Prev.

[17] Francis SS, Wallace AD, Wendt GA (2017). In utero cytomegalovirus infection and development of childhood acute lymphoblastic leukemia. Blood.

[18] Michaelis M, Doerr HW, Cinatl J (2009). The story of human cytomegalovirus and cancer: increasing evidence and open questions. Neoplasia.

[19] Soroceanu L, Cobbs CS (2011). Is HCMV a tumor promoter?. Virus Res.

[20] Herbein G (2018). The human cytomegalovirus, from oncomodulation to oncogenesis. Viruses.

[21] Charlson ME, Pompei P, Ales KL (1987). A new method of classifying prognostic comorbidity in longitudinal studies: development and validation. J Chronic Dis.

[22] Reeves MB, Breidenstein A, Compton T (2012). Human cytomegalovirus activation of ERK and myeloid cell leukemia-1 protein correlates with survival of latently infected cells. Proc Natl Acad Sci U S A.

[23] Buehler J, Carpenter E, Zeltzer S (2019). Host signaling and EGR1 transcriptional control of human cytomegalovirus replication and latency. PLoS Pathog.

[24] de Wit RH, Mujić-Delić A, van Senten JR, Fraile-Ramos A, Siderius M, Smit MJ (2016). Human cytomegalovirus encoded chemokine receptor US28 activates the HIF-1α/PKM2 axis in glioblastoma cells. Oncotarget.

